# Preparation of fish collagen and vancomycin microspheres based on microfluidic technology and its application in osteomyelitis

**DOI:** 10.3389/fbioe.2023.1249706

**Published:** 2023-10-17

**Authors:** Xiaowu Hu, Jinshan Tang, Huaixi Yu, Hanshi Yang, Xiaoqing Lu, Donghui Zheng

**Affiliations:** ^1^ Department of Orthopedics, The Affiliated Huai’an Hospital of Xuzhou Medical University, Huai’an Second People’s Hospital, Huaian, Jiangsu, China; ^2^ Department of Nephrology, The Affiliated Huai’an Hospital of Xuzhou Medical University, Huai’an Second People’s Hospital, Huaian, Jiangsu, China

**Keywords:** fish collagen, vancomycin, microfluidic, osteomyelitis, boon

## Abstract

At present, the clinical treatment of osteomyelitis and osteomyelitis-induced bone defects is challenging, easy to recur, drug toxic side effects, secondary or multiple surgeries, etc. The design of biodegradable composite biomaterials to improve antibiotics in the local precise anti-infection at the same time to complete the repair of bone defects is the current research hot spot. Herein, a composite hydrogel with a double bond at the end (FA-MA) was prepared by affinity addition reaction between fish collagen (FA) and methacrylic anhydride (MA) under photoinitiator initiation conditions, then, FA-MA was amino-activated by EDC/NHC, and vancomycin was attached to FA-MA via amide bonding to prepare FA-MA-Van hydrogels, and finally, the composite hydrogel microspheres were prepared by microfluidic technology. The structure of the hydrogel was confirmed by SEM (elemental analysis), optical microscopy, FTIR, and XPS to confirm the successful preparation. The composite hydrogel microspheres showed the better antimicrobial effect of hydrogel microspheres by bacterial coated plate experiments and SEM morphology results, with the antimicrobial class reaching 99.8%. The results of immunofluorescence staining and X-ray experiments showed that the hydrogel microspheres had a better effect on promoting bone repair. This engineered design of hydrogel microspheres provides clinical significance for treating osteomyelitis at a later stage.

## 1 Introduction

Chronic osteomyelitis is a chronic suppurative inflammation caused by the invasion of pathogenic bacteria into the body and the continuous destruction of bone and surrounding cartilage (El-Hadidi et al., 2018; Masters et al., 2019; Wu et al., 2022). The main routes of infection are blood-borne and traumatic. With the improvement of medical conditions and the quantitative use of antibiotics, patients with chronic blood-borne osteomyelitis gradually decrease. On the contrary, with the rapid development of society, people in high-risk industries such as transportation and construction are prone to fracture, so many internal plants need to be implanted to fix the fracture. The implantation of interior plants increases the infection rate of acute osteomyelitis by 30% (Abulaiti et al., 2022). Without adequate and timely treatment, acute osteomyelitis will gradually evolve into severe chronic traumatic osteomyelitis, whose incidence rate accounts for 80% of osteomyelitis (Zhao and Ferguson, 2020; Wassif et al., 2021; Özkan et al., 2021). Chronic traumatic osteomyelitis is a complex problem in orthopedic treatment due to its complicated condition, long treatment course, uncomplicated fracture, bone defect, poor surgical effect and infection recurrence rate.

The traditional treatment of osteomyelitis is debridement with large doses and the prolonged course of systemic application of antibiotics repeatedly (Cortés-Penfield and Kulkarni, 2019; Metsemakers et al., 2020; Tao et al., 2020). However, due to the poor blood circulation at the focus, the blood drug concentration in bone tissue is less than 20% (Masters et al., 2019; Mirzaei et al., 2020; Nasser et al., 2020), which is difficult to reach the bactericidal concentration. Secondly, reachingthe bactericidal concentration in time is difficult, and the barrier effect of bacterial biofilm is also tricky to effectively kill bacteria (Cobb et al., 2020). In recent years, studies have shown that the refractory infection of osteomyelitis is mainly caused by bacterial biofilm formation (Thonse and Conway, 2007; Jingshu et al., 2018; Jia et al., 2020). The biofilm protects the bacteria from the intervention of antibiotics (Wang et al., 2023), which leads to the “subclinical” state of the focus and is easy to relapse. It is imperative to develop innovative treatment methods. At present, a large number of antibiotic sustained-release materials have been developed to treat osteomyelitis (Hasan et al., 2019; Xu et al., 2021; Feng et al., 2022; Gritsch et al., 2022; Wu et al., 2022), but the treatment effect is still not ideal and the recurrence rate is high. Zhou (Zhang et al., 2023) et al. reported Vancomycin-loaded silk fibroin microspheres in an injectable hydrogel for chronic osteomyelitis therapy, demonstrating a sustained-release profile and good biocompatibility, making it promising for application in osteomyelitis treatment. Ju (Ju et al., 2023) reported a novel Zn^2+^ incorporated composite polysaccharide microspheres for sustained growth factor release and wound healing. The hydrogel microspheres noted above have good value in treating osteomyelitis and wound infection.

Topical antibiotic sustained release systems have emerged as a new therapeutic modality to address this problem better. This treatment modality’s success is mainly because it provides a sustainable and effective bactericidal concentration at the local level of infection, while avoiding the toxicity of drugs associated with systemic antibiotic therapy. Moreover, local antibiotic drug concentrations can exceed systemic intravenous drug concentrations by a factor of fish collagen,a polymeric protein extracted from fish scales that is non-toxic and biocompatible. It is mainly used as a cosmetic material (Trilaksani et al., 2020; Martins et al., 2023). Due to its unique structure, fish collagen can also be used for the preparation of hydrogel microspheres by chemical cross-linking, for the slow release of drugs, and has some bone repair properties (Eap et al., 2014; Zhou et al., 2020; Oh et al., 2021; Furtado et al., 2022). Vancomycin can inhibit the growth and reproduction of bacteria to kill them. It mainly interferes with the peptide, a vital component of the bacterial cell wall structure, thus affecting the formation of the cell wall and inhibiting the production of phospholipids and peptides in the cell wall. It is mainly used in the treatment of drug-resistant bacterial infections, and has a good therapeutic effect on *Staphylococcus aureus*, *Streptococcus* pyogenes, etc. (Kaur et al., 2019; Li et al., 2022; Liu et al., 2022; Xie et al., 2022).

**SCHEME 1:**
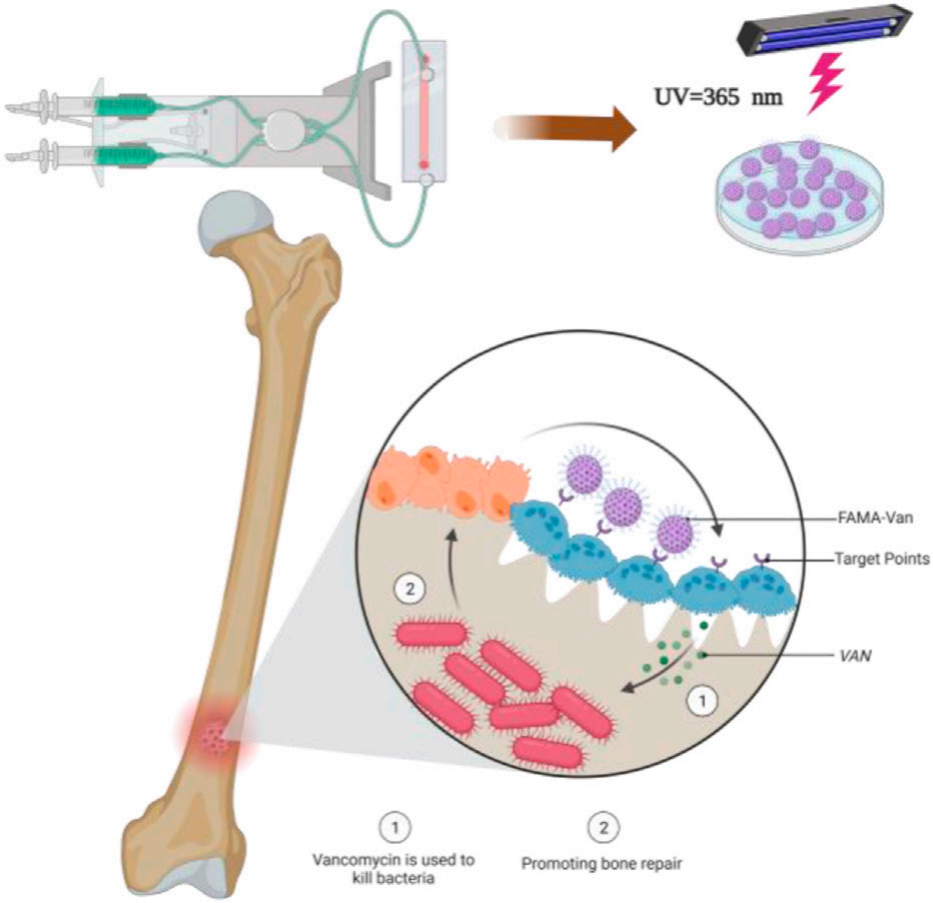
Effect of FA-MA-Van on osteomyelitis focus.

Microfluidics is a science and technology that precisely controls and manipulates micro-scale fluids, with the main characteristics of manipulating fluids in micro-nano-scale space. This technology can prepare monodisperse and uniform hydrogel microspheres (Li et al., 2021; Wang et al., 2021; Illath et al., 2022). The latter is often used as a biodegradable delivery system for carrying and releasing drugs and is widely used in clinical and scientific research fields. In this paper, firstly, methacrylate glycosides were used to modify fish collagen through chemical bonds, and then vancomycin and modified fish collagen were prepared into hydrogel microspheres (FA-MA-Van) by microfluidic technology for the treatment of osteomyelitis. FA-MA-Van can gradually release antibacterial vancomycin at the lesion site, and the degraded fish collagen can also synergistically promote bone repair (Scheme 1).

## 2 Experimental section

### 2.1 Materials

Fish collagen, the methacrylic anhydride were obtained from Acros Co., Ltd. Vancomycin was purchased for Sigma Co., Ltd. Absolute ethanol and all the other reagents were obtained from J&K Co., Ltd. Serum, Bovine Serum Albumin (FBS), Glutaraldehyde was purchased Titan Co., Ltd. Ultra-pure water was utilized throughout this work.

### 2.2 Instrumental

At a resolution of 1 cm^-1^, FTIR spectra were collected from KBr discs at room temperature on Nicolet IS50 spectrophotometer, and 32 scans were performed from 4,000 to 400 cm^-1^.^1^H NMR spectra were obtained on a Bruker 600 MHz instrument. The surface morphologies of the obtained materials were observed by field emission scanning electron microscopy (FESEM). A 1 mg/mL microsphere particle dispersion was prepared, and a few drops of the solution were added dropwise to the silicon wafer. After drying, the sample was fixed on the sample stage with conductive tape for SEM observation. Hydrodynamic particle size testing of different nanomaterials using dynamic light scattering (DLS). Three samples were dispersed in deionized water, filtered and placed in glass vials for testing. Use a Zeta Potential Analyzer to perform Zeta Potential testing on products at various stages in the material synthesis process. Nikon eclipse Ni-U was used to observe the morphology, size and uniformity of cells and microspheres. Bilang Freeze Dryer (FD-2B) is used to freeze dry FA-MA-Van hydrogel, composite hydrogel FA-MA-Van and hydrogel microspheres.

### 2.3 Preparation of FelMA

Mix 30 g of fish collagen and 300 mL of PBS well and stir for 2 h–4 h to completely dissolve the fish collagen, then stir well again in a water bath at 60°C until the fish collagen is completely dissolved and swelled. 16 mL of methacrylic anhydride was slowly added at a rate of 0.25 mL/min using a micro-stirring pump and allowed to react for 2 h. Add 800 mL aliquots of PBS to stop the reaction. Then, the FA-MA solution was transferred to a dialysis bag and dialyzed in deionized water at 38°C for 10 min, and the gel was frozen and dried for 3–4 days using a freeze dryer.

### 2.4 Preparation of FA-MA-Van

Firstly, the carboxy-activator was configured as required (EDC-NHS, 200 mL of water 1.95 g of MES, dissolved and pH adjusted to 5.5, NaCl 2.9 g, NHS 0.69 g, EDC 2.3 g, fully dissolved), followed by adding 10 mg vancomycin and 1 g FMMA to 50 mL of carboxy-activator, and the reaction was carried out at 45°C under stirring The reaction was carried out overnight at 45°C to prepare the product FA-MA-Van, which was covalently linked by carboxyl and amino groups, and the reaction product was dialyzed with deionized water for 3 days. The liquid sample was lyophilized to obtain yellow porous FA-MA-Van, sealed, and stored at −20°C.

### 2.5 Preparation of FA-MA-van microspheres

**SCHEME 2:**
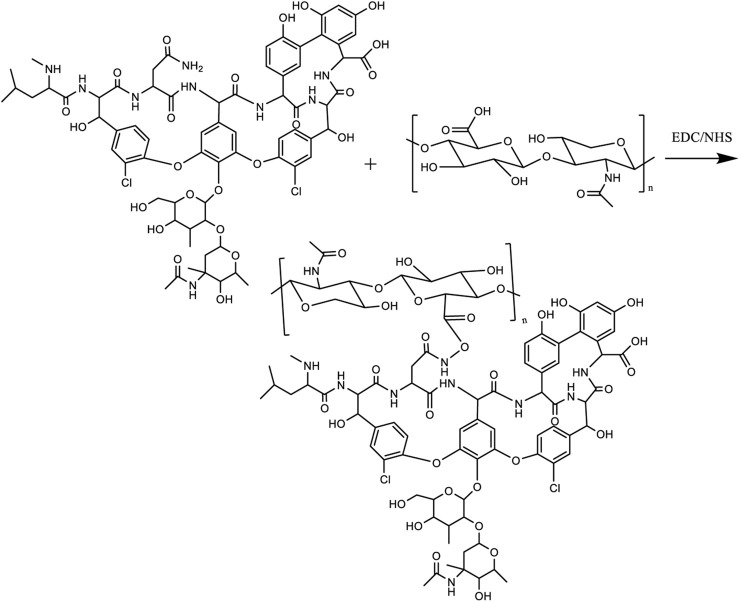
The synthesis of FA-MA-Van compound.

FelMA-Val microspheres were prepared using microfluidic technology. 5% Tween 80 can be used as the microsphere surfactant, and the oil phase is mineral oil to make the shape of the microspheres more stable. Mineral oil was dissolved in deionized water and 0.5% photoinitiator was added. The oil phase can continuously cut off the water, allowing the FelMA-Val to form droplets, which are then transferred from the outlet to the frozen Petri dish. After irradiation under UV light (365 nm, 30 min), FA-MA-Van droplets can be photocrosslinked to form solid gel microspheres. Wash the microspheres repeatedly with 75% ethanol and acetone to remove surfactants and mineral oil. The washed microspheres were kept at −80°C for 3 h, and then FA-MA-Van microspheres were freeze-dried for 72 h (Scheme 2).

### 2.6 Cell culture and cell imaging

Bone mesenchymal stem cells (BMSCs) was used in this study, The cell lines present in this study were obtained from Chinese Academy of Sciences cell bank. Cells were seeded in DMEM, supplemented with 10% fetal bovine serum (FBS) at 37°C in a 5% CO_2_ atmosphere. Then, the cells were cultured in a glass bottom Petri dish having a diameter of 35 mm and allowed to adhere 24 h. Firstly, the cells were washed three times with PBS buffer, FA-MA-Van was added, and incubation was continued for 3 days in a 37°C incubator (5% CO_2_), then the cells were washed three times with PBS, and experimental cells were imaged by inverted fluorescence microscope. Intracellular fluorescence imaging experiments were performed in living cells by means of inverted fluorescence microscopy with × 20 objective lens.

The cytotoxicity of the FA-MA-Van was studied by methylthiazole diphenyltetrazolium bromide (MTT) method. Hela cells grown in log phase were cultured into 48-well plates at a density of 1,000 for 72 h, and cultured at 37°C and 95% air 5% CO_2_ for 12 h, 24 h and 48 h, respectively, and the MTT assay was used as described. Each cytotoxicity experiment was reported three times.

### 2.7 *In vivo* experiments

Twenty-four hours after the mice were injected, the peripheral serum and urine of the mice were collected to detect the content of Alanine aminotransferase (ALT), creatinine (Cr) and creatine kinase (CK) to evaluate the heart, liver and kidney functions of the mice. At the same time, after 7 days of continuous administration, mice were euthanized using carbon dioxide inhalation according to the laboratory animal-guidelines for euthanasia of the People's Republic of China (GB/T 39760-2021). Then mice were dissected, and the heart, liver, spleen, stomach and kidney were taken for HE staining to evaluate the toxicity of the material. Useing anesthesia in our study, Intraperitoneal injection of 10% Chloral hydrate (0.15 ml/10 g).

## 3 Results and discussions

### 3.1 Synthesis and characterization of FA-MA-van

**FIGURE 1:**
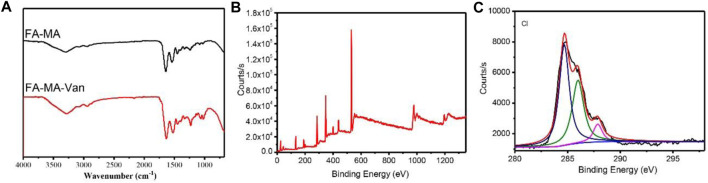
**(A)** Fourier transform infrared spectroscopy of FA-MA and FAMA-Van, **(B)** XPS of FAMA-Van, **(C)** XPS spectrum after Cl peak separation.

FAMA-Van was prepared by using modified fish collagen and vancomycin through microfluidic technology. The structure was characterized by fourier transform infrared, X-ray photoelectron spectroscopy (XPS) and optical microscopy. It can be seen from Figure 1A that for FA-MA, the stretching vibration absorption peak of OH at 3,490 cm^-1^, 2,950 cm^-1^ and 2,867 cm^-1^ are the stretching vibration absorption peaks of alkyl and alkene, 1720 cm-1 is the stretching vibration absorption peak of carbonyl group (C=O), 1620,1587,1432 cm^-1^ is the skeleton vibration absorption peak of benzene ring, 1,105 cm^-1^ is the stretching vibration absorption peak of C-O. For the FAMA-Van microspheres, 3,487 cm^-1^ is the stretching vibration absorption peak of OH, 3,125 cm^-1^ is the stretching vibration absorption peak of -NH_2_, 2,962 cm^-1^ and 2,875 cm^-1^ are the stretching vibration absorption peaks of alkyl and alkene, and 1,643 cm^-1^ is carbonyl group (C=O) stretching vibration absorption peak, 1,604 cm^-1^ is the skeleton vibration absorption peak of C=N double bond, 1,025 cm^-1^ is C-Cl stretching vibration absorption peak and demonstrated that FAMA-Van was prepared successfully. The XPS spectra of FAMA-Van microspheres and XPS spectrum after Cl peak separation were also shown the microspheres was successfully synthesized (Figures 1B, C). Meanwhile, XPS analysis determined confidently the chemical composition of FA-MA-Van hydrogel microspheres.

**FIGURE 2:**
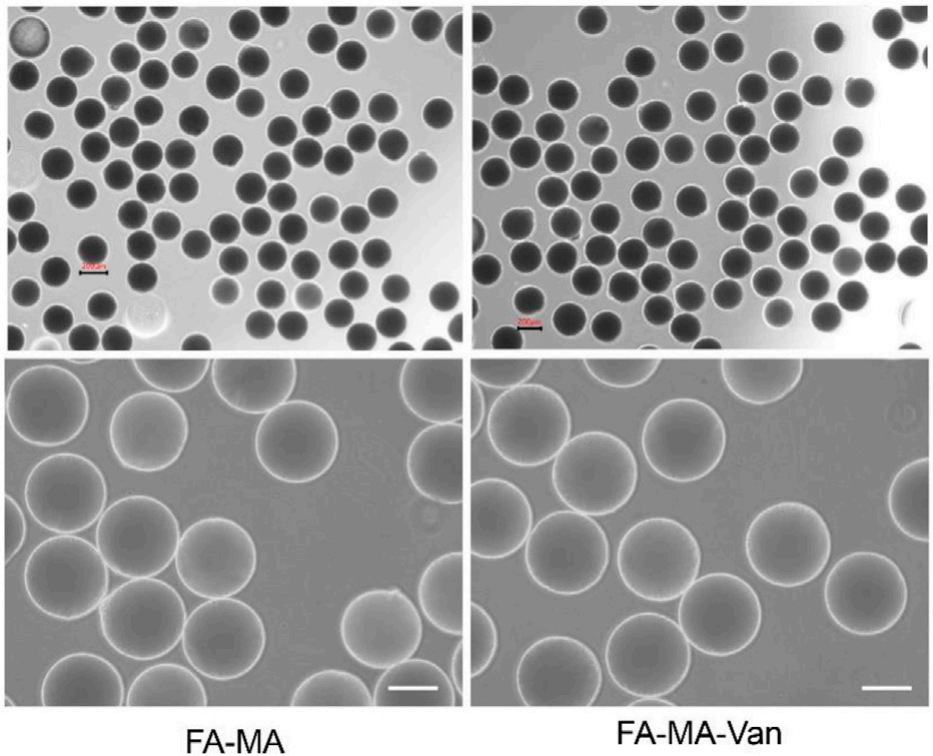
Optical microscope images of FA-MA and FA-MA-Van.

**FIGURE 3:**
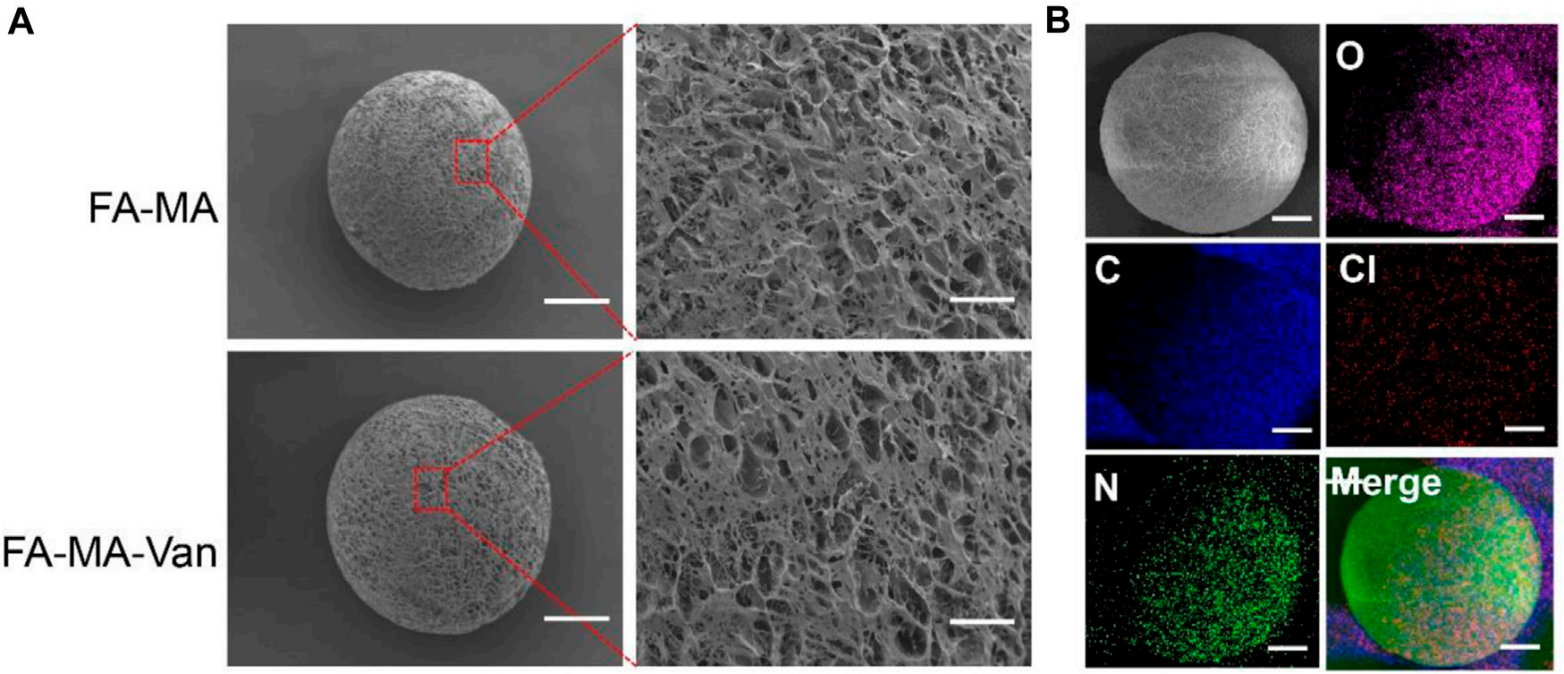
**(A)** SEM image and element distribution, **(B)** The mapping spectrum analysis of hydrogel microspheres, Scale: 50 μm.

Figure 2 was a micrograph of the composite microspheres swelled in PBS solution. The hydrogel microspheres have good morphology and are relatively uniform. Figures 3A, B showed the overall and partial magnified images of the lyophilized FA-MA and FA-MA-Van microspheres by scanning electron microscope (SEM). Scanning electron microscopy observed that the freeze-dried microspheres were porous. In addition, SEM showed that the average particle size of FA-MA-Van microspheres is 200 ± 15 nm. Therefore, energy dispersive X-ray spectroscopy (EDS) showed that FA-MA-Van microspheres had uniform C, N, O, Cl element distribution and FA-MA-Van microspheres had uniform magnesium element distribution. At the same time, the release curve of PBS and FA-MA-Van was tested, and the results showed that the drug reached its highest value within 4 days and could last for 25 days.

### 3.2 Cytocompatibility and proliferation evaluation of FA-MA-van

**FIGURE 4:**
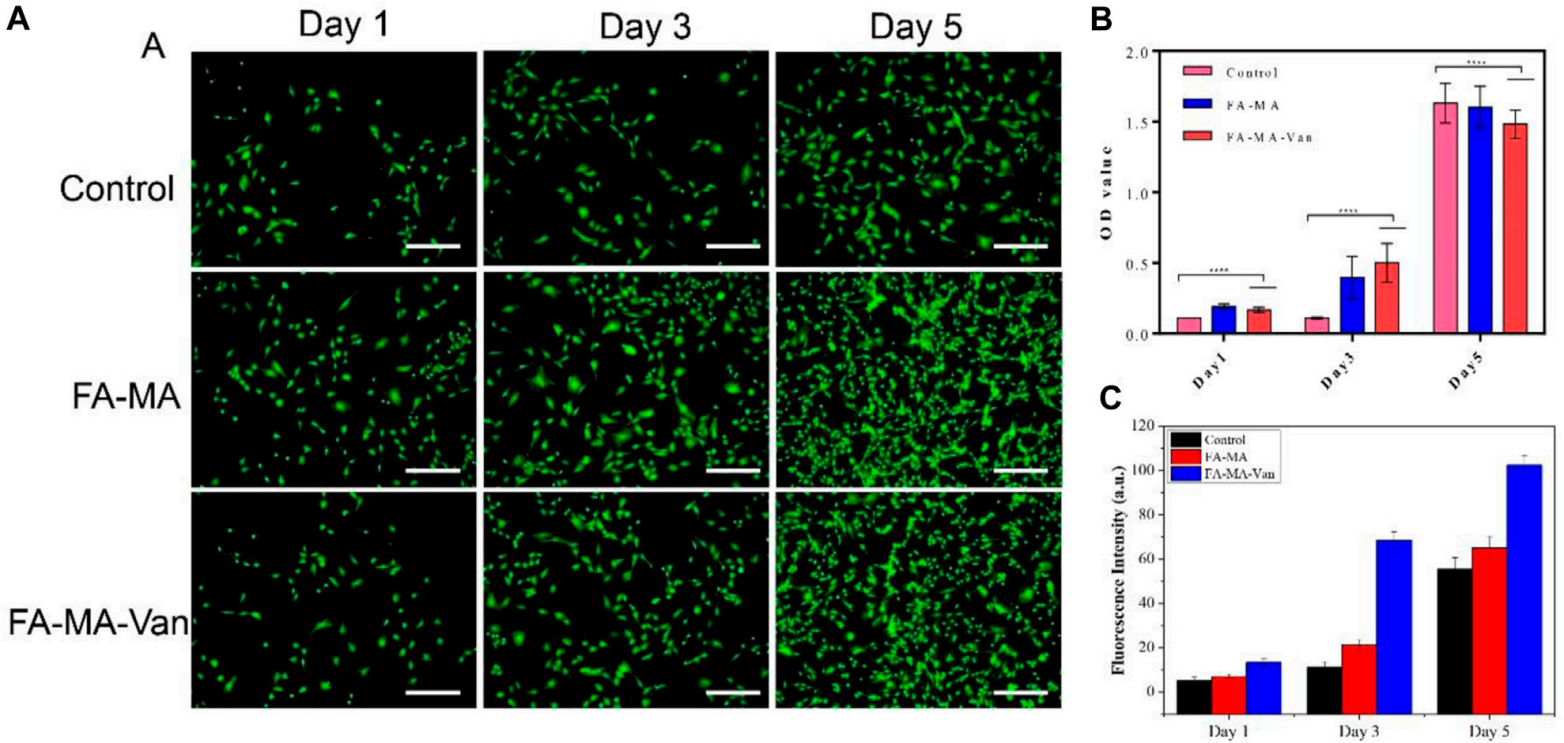
**(A)** Representative fluorescence images of control, FA-MA and FA-MA-Val co-cultured BMSCs by live-dead staining assay; **(B)** Cell proliferation of control, FA-MA and FA-MA-Van; **(C)** Fluorescence statistics of control, FA-MA and FA-MA-Van.

In order to verify the cytotoxicity of FA-MA Val hydrogel microspheres on bone marrow mesenchymal stem cells (BMSCs) *in vitro*, PBS, FA-MA and 1 *μ* M FA-MA-Van and BMSCs were co-cultured, and the cultured cells were subjected to live death staining (Figure 4A), CCK-8 detection (Figure 4B) and cell fluorescence counting analysis (Figure 4C) on the 1st, 3rd and 5th day respectively. The results showed that the cell density of the three groups increased with the increase of culture time, and almost all the cells in each group were alive, without obvious dead cells. Compared with the control group, the cell proliferation activity of FA-MA group and FA-MA-Van group were significantly different at each time point, and the cell proliferation activity of FA-MA-Van group was much higher than that of the previous two groups, showing an amazing proliferation-promoting ability. FA-MA group did not promote cell proliferation as FA-MA-Van group, which may be due to the fact that the microsphere and cell co-culture did not release antibacterial vancomycin. In general, the above results show that the materials in this study have good biocompatibility with BMSCs and can effectively promote cell proliferation.

**FIGURE 5:**
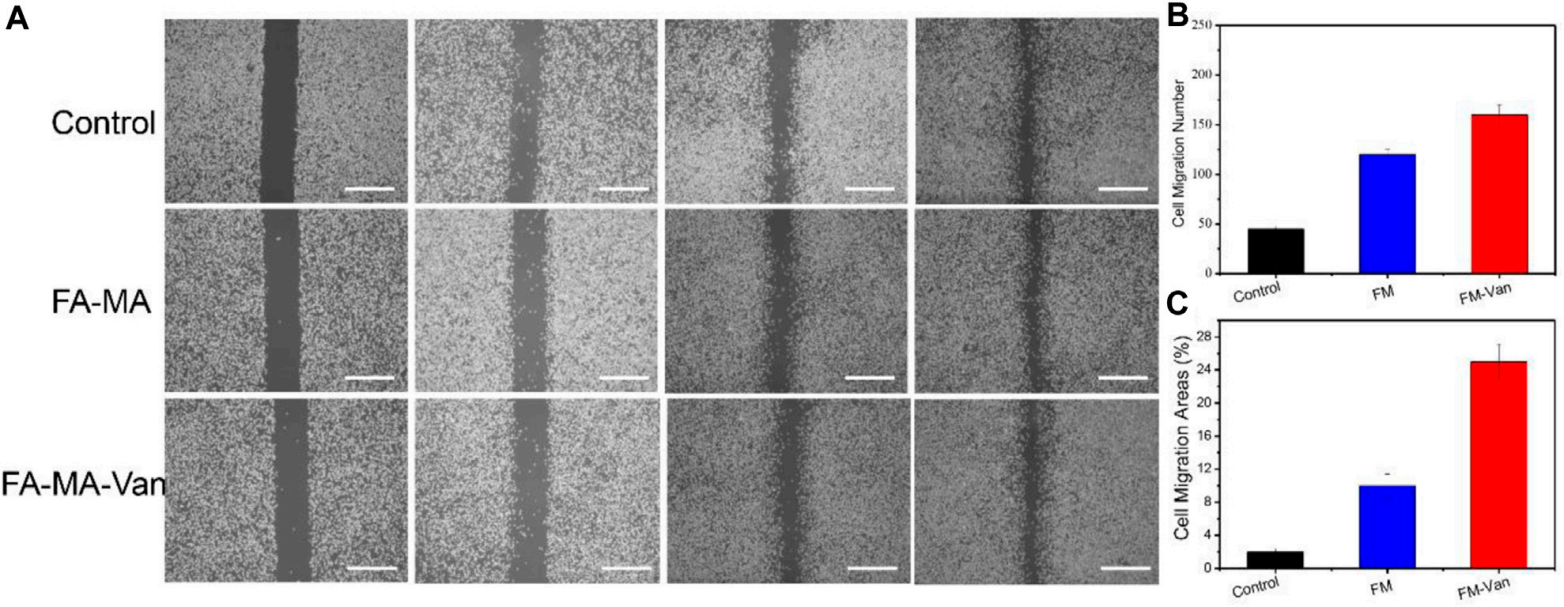
**(A)** BMSCs cell scratch test of FA-MA and FA-MA-Van with concentration 1uM; **(B)** The number of cell migration in cell scratch, **(C)** Cell migration region in cell scratch, scale: 50 μm.

In order to further verify the proliferation effect of FA-MA Val hydrogel microspheres on BMSCs cells, the cell scratch test was tested. First, BMSCs cells were inoculated and cultured in a 12-well plate, and the tip of the pipette was scratched to form a wound. Then add the medium containing different ingredients to culture for 0 h, 12 h, and 24 h. It can be seen from Figure 5 that after 24 h of culture, FA-MA-Van has obvious migration wounds. In contrast, the wound area of FA-MA-Val group is 14.50%, significantly lower than that of other groups. (This is the use of scratches to simulate the wound at the cell level) This shows that FA-MA-Van microspheres have the proliferation effect on BMSCs, which is very consistent with the cell compatibility.

### 3.3 Antibacterial properties of FA-MA-van

**FIGURE 6:**
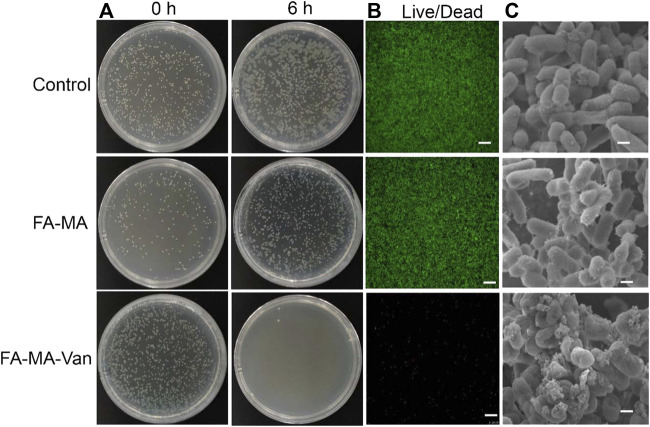
FA-MA-Val antibacterial test. **(A)** Optical image of *Staphylococcus aureus*. **(B)** Live-death staining image of bacteria. **(C)** SEM image of bacteria. Scale: 50 μm.

After incubating the bacterial solution on the hydrogel material overnight, take out 200 μL solution, drop it on the solid medium, apply it to the plate and incubate it at constant temperature overnight, and observe the surface bacterial colony. The results showed (Figure 6A) that the number of colonies in the hydrogel containing Van was less than that in the hydrogel without Van. The colony number of *Staphylococcus aureus* gradually decreased with the increase of Val release. There was no obvious *Staphylococcus aureus* on the agar plate surface after 6 h of release. This result confirmed that FA-MA-Val has antibacterial effect. It is further shown by the live and death staining of bacteria (Figure 6B) that when the bacteria are cultured with FA-MA-Van, all the bacteria have died. This phenomenon is consistent with the above, which indicates that FA-MA-Val has antibacterial effect.

In order to further study the mechanism of bacterial growth in FA-MA-Van, the bacterial morphology was characterized. SEM results (Figure 6C) show that when *Staphylococcus aureus* is cultured on the hydrogel without Val, it presents a typical bacterial shape of complete spherical and rod-shaped structure. In contrast, when bacteria are cultured on FA-MA-Van hydrogel, the bacterial colony and structural integrity are greatly affected, and the bacterial colony is small or collapses. The results showed that FA-MA-Van significantly enhanced the antibacterial effect of the hydrogel.

### 3.4 Promote bone repair of FA-MA-van

**FIGURE 7:**
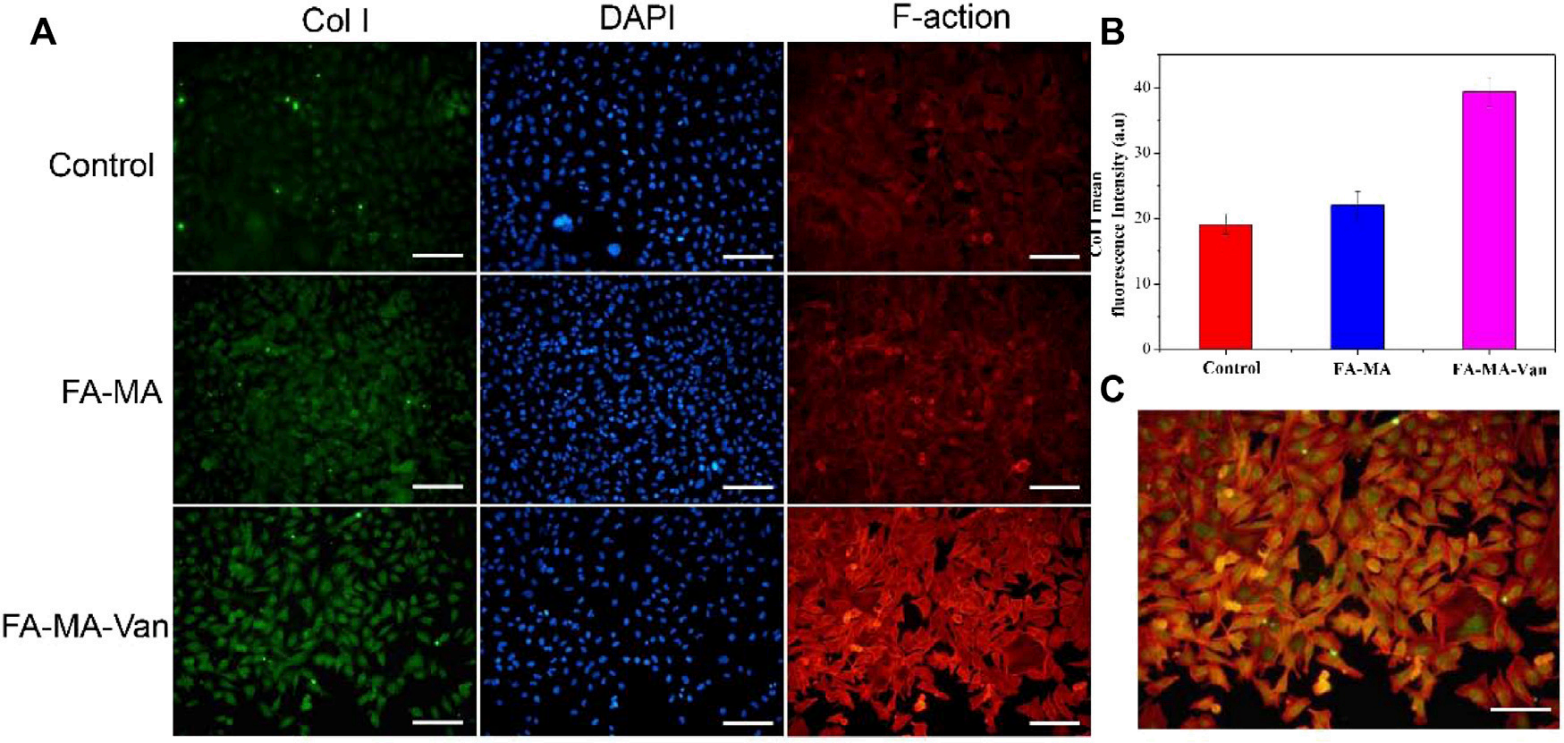
**(A)** Immunofluorescence images of BMSCs. **(B)** The fluorescence quantification of Col 1 collagen. **(C)** Merge diagram of red, blue, and green channels in the FA-MA-VAN group. Scale: 50 μm.

**FIGURE 8:**
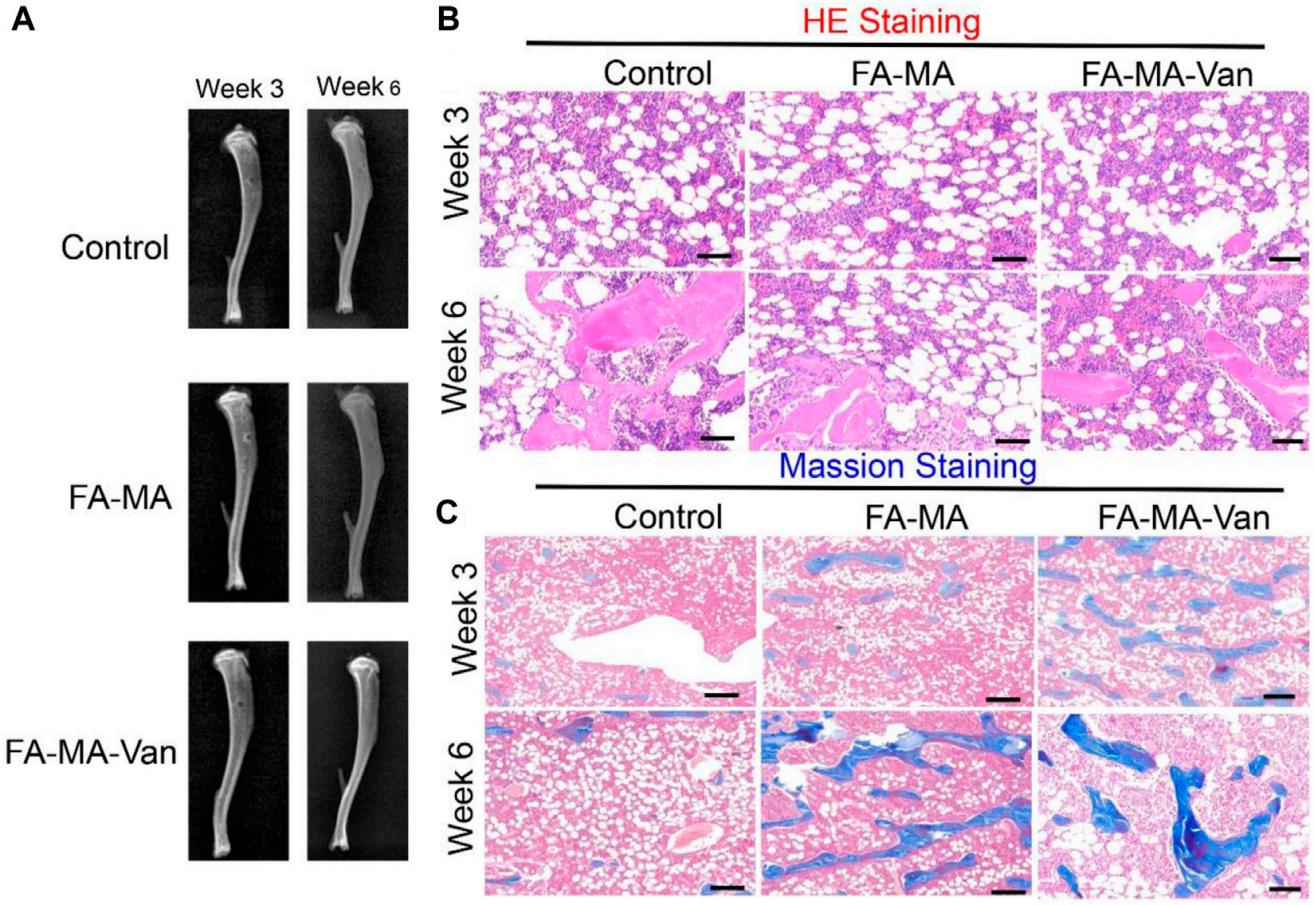
**(A)** X-ray image of mice week 3 and week 6; The **(B)** the HE staining **(C)** the massion staining for control, FA-MA and FA-MA-van with week 3 and week 6. Scale: 50 μm.

It was verified by immunofluorescence that FA-MA-Van had a promoting effect on bone formation of BMSCs. TGF-β 3 is a crucial growth factor in inducing chondrogenic differentiation of stem cells, and also an important component in the chondrogenic induction medium of stem cells *in vitro*. It is widely used in various cartilage regeneration engineering scaffolds. However, the harsh storage conditions and high market prices of growth factors hinder their widespread application in clinical practice. This study aims to replace TGF-β 3 with fish collagen with more economic advantages and more stable properties for cartilage induction. The solution of FA-MA-Van was used for the induction and culture of BMSCs. Immunofluorescence staining was performed on the induction results to show the expression level of type I collagen in the cell mass (Figure 7A). In addition, DAPI and F-action staining were also performed on BMSCs cells. From Figure 7A, it can be found that the expression level of type I collagen in FA-MA-Van group is significantly higher than that in other groups, and the cytoskeleton and nucleus are damaged. It shows that it can promote osteogenesis. *In vivo* experiment, the rat osteomyelitis model was first established, and PBS, FA-MA and FA-MA-Van were injected into the joint cavity at the second week after operation. X-ray imaging of knee joint was performed at the 1st and 6th week after operation (Figure 8A). The HE staining and Masson Staining displayed the FA-MA-Van hydrogel microspheres can better repair osteomyelitis. It can be seen from the figure that FA-MA-Van group had the best recovery of focus.

## 4 Conclusion

In conclusion, FAMA-Val microspheres were grafted with Van through condensation reactivity and ligand bonding. Thus, functional microfluidic FAMA-Val microspheres were constructed to endow hydrogel microspheres with active trapping, minimally invasive injection and continuous sustained release of Van, thus enabling antimicrobial effects as well as enhancing their ability to activate osteoblasts and endothelial cells. Apparently, grafting Val on FAMA microspheres gives FAMA-Val microspheres strong bacterial trapping and sustained release properties. In vitro experiments, FAMA-Val composite microspheres showed excellent bacterial capture and osteogenic potential, and X-ray experiments further demonstrated that FAMA-Val composite microspheres could promote regeneration of bone defects in rats, and bacterial culture experiments were able to kill bacteria indicating good antibacterial effect. The cell survival rate was higher than 80% even at high concentrations for 48 h, and the cytocompatibility was particularly good. The data from HE staining demonstrated that the material was not toxic *in vivo*. More importantly, the material has an excellent alleviating effect on osteomyelitis and an effective antibacterial effect.

## Data Availability

The original contributions presented in the study are included in the article/Supplementary Material, further inquiries can be directed to the corresponding author.
