# Supplementary material for: Preparation of fish collagen and vancomycin microspheres based on microfluidic technology and its application in osteomyelitis
Source: Front Bioeng Biotechnol. 2023 Oct 17;11:1249706. doi: 10.3389/fbioe.2023.1249706 (PMC10616836; doi:10.3389/fbioe.2023.1249706)
Supplement: Supplementary file 1 [file DataSheet1.docx]

**Supporting Information for**

**Preparation of fish collagen and vancomycin microspheres based on microfluidic technology and its application in osteomyelitis**

*Xiaowu Hu^1^, Jinshan Tang^1^, Huaixi Yu^1^, Hanshi Yang^1^, Xiaoqing Lu^1^, Donghui Zheng^2^**

1 Department of Orthopedics, Huai'an Second People’s Hospital, The Affiliated Huai'an Hospital of Xuzhou Medical University, Huai'an,jiangsu，223002，P.R.China

2 Department of Nephrology, Huai'an Second People's Hospital, The Affiliated Huai'an Hospital of Xuzhou Medical University, Huai'an,jiangsu，223002，P.R.China

Corresponding author: [zddwjj@126.com](mailto:zddwjj@126.com)

***Cell culture and cell imaging***

cells were seeded in DMEM, supplemented with 10% fetal bovine serum (FBS) at 37°C in a 5% CO_2_ atmosphere. Then, the cells were cultured in a glass bottom petri dish having a diameter of 35 mm and allowed to adhere 24 h. Firstly, the cells were washed three times with PBS buffer, FA-MA-Van was added, and incubation was continued for 3d in a 37 °C incubator (5% CO_2_), then the cells were washed three times with PBS, and experimental cells were imaged by inverted fluorescence microscope. Intracellular fluorescence imaging experiments were performed in living cells by means of inverted fluorescence microscopy with 20×objective lens.

The cytotoxicity of the FA-MA-Van was studied by methylthiazole diphenyltetrazolium bromide (MTT) method. Hela cells grown in log phase were cultured into 48-well plates at a density of 1000 for 72 h, and cultured at 37 °C and 95% air 5% CO_2_ for 12 h, 24 h and 48 h, respectively, and the MTT assay was used as described. Each cytotoxicity experiment was reported three times.

Figure S1 The in vitro release curve of PBS and FA-MA-Van
